# Faecal glucocorticoid metabolites as a measure of adrenocortical activity in polar bears (*Ursus maritimus*)

**DOI:** 10.1093/conphys/coaa012

**Published:** 2020-04-04

**Authors:** Anna Hein, Rupert Palme, Katrin Baumgartner, Lorenzo von Fersen, Benno Woelfing, Alex D Greenwood, Thea Bechshoft, Ursula Siebert

**Affiliations:** 1 Institute for Terrestrial and Aquatic Wildlife Research (ITAW), University of Veterinary Medicine Hannover, Bischofsholer Damm 15, 30173 Hannover, Germany; 2 Unit of Physiology, Pathophysiology, and Experimental Endocrinology, De for Biomedical Sciences, University of Veterinary Medicine Vienna, Veterinärplatz 1, 1210 Vienna, Austria; 3 Nuremberg Zoo, Am Tiergarten 30, 90480 Nuremberg, Germany; 4 Department of Wildlife Diseases, Leibniz-Institute for Zoo and Wildlife Research, Alfred-Kowalke-Strasse 17, 10315 Berlin, Germany; 5 Department of Veterinary Medicine, Freie Universität Berlin, Oertzenweg 19b, 14163 Berlin, Germany; 6 Department of Bioscience, Aarhus University, Frederiksborgvej 399, 4000 Roskilde, Denmark

**Keywords:** biological validation, EIA, faecal glucocorticoid metabolites, non-invasive HPA axis assessment, stress, *Ursus maritimus*

## Abstract

Analysis of faecal glucocorticoid metabolites (FGMs) is frequently applied to assess adrenocortical activity in animal conservation and welfare studies. Faecal sample collection is non-invasive and feasible under field conditions. FGM levels are also less prone to circadian rhythms, episodic fluctuations and short acute stressors than glucocorticoid (GC) levels obtained from other matrices, for example blood or saliva. To investigate the suitability of FGM measurement in polar bears (*Ursus maritimus*), a species listed as Vulnerable by the IUCN (International Union for Conservation of Nature), a cortisol enzyme immunoassay (EIA) was biologically validated by demonstrating a significant increase in FGMs after five zoo-to-zoo transports. In addition to validating the method, the study also documented an average delay of 7 h until the first occurrence of food colorants in the monitored polar bears, which provides essential information for future studies. After validation, the assay was applied to measure FGM concentrations of five polar bears over a 1-year period. Several pre-defined potentially stressful events were recorded in an event log to measure their effect on FGM concentrations. A mixed model analysis revealed significant increases in FGM concentrations after social tension and environmental changes, whereas season and sex had no significant effect. The study demonstrates that the applied cortisol EIA is suitable for measuring FGM levels in polar bears and that using a carefully validated assay for FGM analysis in combination with a detailed sampling protocol can serve as a valuable tool for evaluating mid- to long-term stress in polar bears. FGM levels can be used to monitor stress in captive polar bears in order to optimize housing conditions but also to elucidate stress responses in wild populations for targeted conservation measures.

## Introduction

The analysis of glucocorticoids (GCs) has been widely used for determining stress levels of a range of domesticated and wild animals (for a detailed list, see Sheriff *et al.*, 2011)*.* Stress is most commonly defined as an imbalance of homeostasis in response to external stimuli, called stressors. Stressors can be of various nature: any physical and/or psychological occurrence perceived by the organism can evoke a stress response in order to restore homeostasis. Under acute stressful conditions, GCs—in most mammals mainly cortisol (Harlow *et al.*, 1990; Hadley, 1996)—are released rapidly from the adrenal glands into the blood. Although fast activation of the hypothalamic–pituitary–adrenal (HPA) axis is part of a natural and vital defence mechanism, prolonged elevated GC levels may lead to negative health consequences for an individual, e.g. immuno-suppression, reduced growth and reproduction (Sapolsky *et al.*, 2000; Habib *et al.*, 2001; McEwen, 2007)*.* Furthermore, in chronically stressed individuals negative feedback loops can be disrupted which can lead to HPA axis dysregulation (Dantzer *et al.*, 2014)—though there are species that show no adverse effects (in the long term) or adrenal exhaustion under chronic stress (Boonstra, 2013). How and whether a species responds to changing factors (stressors) in its environment and thus can sustain itself long-term is the main criterion defining its health (Patyk *et al*., 2015). Therefore, by measuring GC levels, stress responses can be evaluated and important stressors identified, e.g. in the context of housing criteria and captive animal welfare as well as ecological changes and their impact on free-ranging animals (Möstl *et al.*, 1999; Millspaugh and Washburn, 2004; Mormede *et al.*, 2007; Sheriff *et al.*, 2011; Palme, 2012; Dantzer *et al.*, 2014).

GCs can be measured in a range of sample types, each holding advantages and disadvantages regarding invasiveness of sampling methods and time spans represented by the measured GC levels (see reviews by Möstl and Palme, 2002; Mormede *et al.*, 2007; Sheriff *et al.*, 2011; Palme, 2019). However, even in ‘non-stressed’ individuals, the physiological secretion of GCs occurs pulsatile in most mammals and circadian variation of plasma and salivary GC levels can be observed*.* Thus, GCs measured in blood or saliva samples represent point estimates reflecting only short time frames (Kirschbaum and Hellhammer, 1989; Romero and Reed, 2005). Also, bias caused by stress-evoking handling or restraint situations, as for example during blood collection, should be avoided (Cook *et al.*, 2000; Romero and Reed, 2005; Sheriff *et al.*, 2011). Contrary to other matrices, faeces can be easily collected from the ground in captive and natural contexts. GCs are quickly metabolized in the liver, and their metabolites are excreted via the bile into the gut. Therefore, cortisol itself or other native GCs cannot be found in faeces in any meaningful quantities, which is why their metabolites are used instead for an integrated measurement of adrenocortical activity (Möstl and Palme, 2002; Ganswindt *et al.*, 2003; Palme, 2005; Touma and Palme, 2005; Palme, 2019). Another advantage of faecal glucocorticoid metabolite (FGM) analysis is its long-term character: FGMs reflect the average free (unbound) blood GC levels over several hours (6–24 h, depending on the species), and thus fluctuations due to secretory patterns are mitigated (Palme *et al.*, 2005; Sheriff *et al.*, 2011; Shepherdson *et al.*, 2013; Palme, 2019).

Before applying FGM analysis, species-specific assay validation is essential. To determine if HPA axis activity is well reflected in FGM levels, stimulation or suppression of the adrenal cortex can be performed. In free-ranging or less accessible animals, a biological validation is often conducted: the effect of a known stressful event, e.g. capture or transport, is monitored by analysing serial samples before and after the occurrence of the stressor (Touma and Palme, 2005; Palme, 2019). The species-specific time delay between elevated plasma GC levels and FGM excretion also needs to be evaluated before application of FGM analysis to be able to temporally correlate measured levels.

FGMs have been measured using various radioimmunoassays (RIAs) in polar bears (Shepherdson *et al.*, 2004; Shepherdson *et al.*, 2013; White *et al.*, 2015). Only one study used an enzyme immunoassay (EIA) in this species, though with a non-validated assay (Bryant and Roth, 2018). Even though RIAs offer some benefits, including long-time application and high precision, there are several disadvantages in their utilization such as risk of radiation exposure for staff and therefore the need for specialized laboratories and toxic waste disposal (Möstl *et al.*, 2005; Yadav *et al.*, 2013).

The aim of the current study was to first validate a more practicable EIA for analysing polar bear FGMs. In a second step, the validated assay was applied to determine individual long-term FGM levels in the context of seasonal variation and cause–effect relations to understand polar bear HPA axis activity. Measuring FGMs from polar bears could be a valuable diagnostic tool for the long-term assessment of health and well-being of both zoo and free-ranging polar bears. Quantifying the impact of human disturbance and specifying the most important factors for polar bear populations could help guide conservation efforts.

## Materials and methods

### Subjects and study design

#### Study animals and facilities

Faecal material was collected from a total of eight zoo-housed polar bears in Europe (Table 1). The 12 participating zoos were all members of the European Association of Zoos and Aquaria (EAZA), and polar bears were kept under comparable conditions (e.g. similar diet, predominant use of outdoor enclosures). Females were presumably non-pregnant (no signs of a birth/stillbirth, no withdrawing into the provided den; transported females were sexually immature); they were neither lactating nor carrying offspring during the entire period of the study. Each zoo received a starter kit with the necessary material, detailed sampling instructions and protocols for recording sampling conditions (including date/time, known/estimated age of sample, diarrhoea, temperature range/weather, inside/outside location; see Supplementary Material). Furthermore, zoo keepers responsible for the polar bears were interviewed with the help of a questionnaire to obtain information on management practices, daily husbandry routines and feeding (see Supplementary Material).

**Table 1:** Overview of polar bears and faecal samples collected during transportation events and long-term sampling

Polar bear	Sex	Reproductive status	Age (years^a^)	Number of experienced transportation events^b^	Number of faecal samples			Excluded samples (older than 12 h^f^)	Otherwise excluded samples^g^	Total sample number
					Pre-transport (days^c^)	Post-transport (days^d^)	Long-term (months^e^)			
A	m	Fertility proven	12	7	9 (22)	11 (33)	123^h^ (22)	11	4	108
					6 (5)	8 (23)				
B	m	Fertility proven	16	5	25 (52)	22 (33)				47
C	f	Sexually immature	1	1	9 (8)	13 (15)				22
D	f	Sexually immature	2	1	6 (6)	13 (16)				19
E	m	Fertility unknown	5	1			34 (14)	4	1	29
F	m	Infertility proven	7	2			47 (12)			47
G	f	Non-pregnant^i^/fertility unknown	5	1			34 (14)	3	2	29
H	f	Non-pregnant^i^/fertility unknown	11	2			23 (7)		1	22

^a^Age at start of sampling phase

^b^Including monitored transports

^c^In parenthesis: number of sampling days before transport

^d^In parenthesis: number of sampling days after transport

^e^In parenthesis: number of consecutive sampling months

^f^Time between defaecation and sample collection, according to sampling protocol

^g^Missing sampling protocols and/or sample labels

^h^Including transport samples

^i^Presumed non-pregnancy based on lack of behavioural signs (e.g. withdrawing into the den) or other evidence such as birth/stillbirth

#### Pilot study: gastrointestinal transit time in polar bears

Gastrointestinal transit time (GTT) in polar bears was determined by adding food colorants (article number 2110 red, article number 2112 green, Brauns-Heitmann, Warburg, Germany) to the morning feed (beef, chicks, capelin, mackerel, whiting, dog food, cucumber, salad, carrots, fruits and egg in varying proportions) of one female and one male polar bear in Zoo Karlsruhe during late summer (when food was taken up at least at 4 days per week). The first occurrence of coloured scats was recorded (total number of trials = 5).

#### EIA validation

For the biological validation of the assay, faecal samples were collected from four bears (two males, two females) that were translocated from one zoo to another (five transports, one male was transported twice). Translocations occurred between 2015 and 2017 according to breeding recommendations of the European Endangered Species Programme (EEP; Szánthó and Schad, 2014). Transport and the activities related to it have been shown to be stressful for various animals, i.e. led to a rise of FGM concentrations (Palme, 2005; Touma and Palme, 2005; Shepherdson *et al.*, 2013). We therefore expected FGM levels to increase soon after a transportation event in polar bears. Transport-related stress can be measured by a suitable assay—i.e. an assay which provides significant differences between pre- and post-stressor FGM levels (Möstl *et al.*, 2005; Palme, 2019). Faecal sampling started at least 5 days prior to transport, continued during transport and lasted for at least 1 week after transport. As many fresh faecal samples as possible were collected (<24 h; for sample numbers, see Table 1), and a simple sampling protocol was filled out by the keepers (see Section 2.1.1). Feeding regimes on transportation days did not change from the usual routine according to keepers. All five monitored transports took place over a single day (transportation time: 3–8 h); two of the four polar bears (B, C; Table 1) had to be immobilized before transport (using 2.1 mg/kg zolazepam/tiletamine (Zoletil® 100, Virbac S.A., Carros, France) + 0.04 mg/kg medetomidine (Zalopine® 30 mg/ml, Orion Corporation, Espoo, Finland) and 0.2 mg/kg atipamezole (Antisedan® 5 mg/ml, Orion Corporation) for reversal).

#### Longitudinal measurements of faecal GC metabolites

In order to establish individual FGM long-term profiles, faecal material was gathered for an average of 13 consecutive months (range: 7–22 months) from three adult males and two adult female polar bears in five different zoos (one male was transported repeatedly during the monitored time, three different zoos participated in sampling of this bear and pre- and post-transport samples were part of the assay validation; Table 1). Keepers collected one to three faecal samples per week per bear. In addition to the standard sampling protocol (see Section 2.1.1), pre-defined stress-inducing events (‘fight’, ‘mating’, ‘socialization’, ‘separation’, ‘change of enclosure’, ‘environmental changes’, ‘disease’ and ‘other’ (free text)) were recorded in an event log parallel to the sampling by the keepers to take cause-effect relations into account (see Supplementary Material).

### Sample collection, processing and extraction

Faecal samples were collected by the keepers during the usual daily cleaning routine of the bears’ outdoor or indoor areas without disturbing them. If more than one sample was collected within the same day, each sample was analysed separately and the mean FGM concentration determined. If more than one bear was kept in the same enclosure, individual bears were fed with food colorants (see Section 2.1.2), beetroot or maize to differentiate scats from different individuals.

Faecal material older than 24 h was avoided to reduce the risk of microbial degradation and subsequent changes in FGM levels (Möstl *et al.*, 1999; Möstl and Palme, 2002; Beehner and Whitten, 2004; Millspaugh and Washburn, 2004). Faeces that was still moist on the surface was estimated to have been excreted within a 12-h period (author’s personal experience and communication with zoo keepers (T. Ramm, M. Ehlers, head polar bear keepers, Karlsruhe Zoo); older/dry samples were marked in the protocol). Faecal material from outdoor enclosures exposed to extreme weather like heavy rain or temperatures above 25°C was excluded from analysis to avoid changes in FGM concentrations due to environmental factors (exclusion based on external appearance or sampling protocols, respectively; Möstl *et al.*, 1999; Khan *et al.*, 2002; Terio *et al.*, 2002; Touma and Palme, 2005). Each sample was mixed until homogeneous as FGMs are not evenly distributed within boli (Palme *et al.*, 1996; Millspaugh and Washburn, 2004). Subsequently, hen egg size subsamples were transferred to labelled plastic sample bags. Immediately after collection, the samples were frozen and stored by the participating zoos (at −20°C) in order to prevent degradation by bacterial enzymes (Möstl and Palme, 2002; Beehner and Whitten, 2004). On completion of sampling, frozen faecal material was transported via overnight express (packed with Styrofoam and freezer packs to prevent thawing) to the laboratory of the Veterinary University of Vienna (Unit of Physiology, Pathophysiology and Experimental Endocrinology, Department for Biomedical Sciences) and kept at −20°C in the freezer until further processing.

The steroid extraction procedure was performed at the Veterinary University of Vienna as described by Palme *et al.* (2013). Briefly, samples were defrosted at 40°C for 20 min and mixed again and 0.5 g of the wet faeces was weighed into test tubes after removing undigested material (e.g. maize, bones). Immediately afterwards, 4 ml methanol 100% (No. 1.06009.2500, Merck, Darmstadt, Germany) and 1 ml double-distilled water (≙ 5 ml 80% methanol) were added, as this protocol has yielded the best results for steroid extraction in nearly all mammalian species previously tested (Schatz and Palme, 2001; Touma and Palme, 2005; Palme *et al.*, 2013). By keeping the time between thawing and addition of the organic solvent short, further degradation after thawing could be prevented since deep-freezing alone does not eliminate bacteria which enzymatically degrade steroids (Möstl *et al.*, 2005).

After thoroughly shaking for 30 min at 20°C (RapidVap 7900001, Labconco, Kansas City, MO, USA), samples were vortexed for 30 s (Minishaker MS1, IKA, Staufen, Germany) and centrifuged at 2500 g for 12 min at 20°C (CS-6KR Centrifuge, Beckman, Indianapolis, IN, USA). Fifty microlitres of the supernatant was then pipetted into microtubes, diluted with 450 μl assay buffer and stored at −20°C until further analysis.

### Faecal GC metabolite analysis

In a first step, three EIAs were tested, a cortisol EIA (Palme and Möstl, 1997), an 11-oxoaetiocholanolone EIA (Möstl and Palme, 2002) and a 5α-pregnane-3β,11β,21-triol-20-one EIA (Touma *et al*., 2003) on a subset of samples (transport event in a single male/female bear). Peak increases (percentage above baseline) of measured FGM levels were highest for the cortisol EIA (male/female: 2013%/208% compared to 1510%/94% and 1080%/108% for the other two EIAs, respectively). Thus, all samples were only analysed with the cortisol EIA, which has previously been successfully applied in other carnivores such as dogs, wolves and aardwolves (Palme *et al.*, 2001; Schatz and Palme, 2001; Ganswindt *et al.*, 2012; Molnar *et al.*, 2015). The EIA measures FGMs with a 11ß,17α,21-triol-20-one structure (for details including cross-reactions, see Palme and Möstl, 1997). Serial dilutions of extracts containing high FGM concentrations (one for each sex) were performed and yielded curves parallel to the standard curve. Intra-assay coefficients of variation (CVs; 10 repetitions each) for a high- and low-concentration pool sample were 5.9 and 4.2%. Inter-assay CVs (30 repetitions each) were 9.7% (high) and 12.5% (low). All samples were analysed on 15 plates in total.

### Data analysis

#### EIA validation

In order to test if the FGM levels (in ng/g faeces) after a transportation event differed from those before the transportation event, pre- and post-transportation time windows were defined. The pre-transportation time window included the 5 days before transportation, being the maximum period of time for which samples were available in all five transports. The post-transportation time window was Day 1 to 6 after the transportation event to cover not only the transportation event itself but also other possible stressful events related to transport (e.g. new enclosure). Since FGM levels were non-normally distributed, the median FGM level was determined for each individual and time window and a one-sided paired Wilcoxon signed-rank test was used to test if the median FGM levels in the post-transportation window had increased relative to the levels in the pre-transportation window. Finally, a Kruskal–Wallis rank-sum test was used to analyse if FGM levels in the 5-day pre-transportation window differed significantly between individuals.

To estimate effect sizes and to characterize the timing of the increase in the FGM levels after a transportation event, baseline FGM concentrations before transport were calculated for each bear individually using an iterative approach as suggested by Palme (2019). Thus, pre-transport FGM values higher than the mean plus two standard deviations (SD) were excluded until all values fell within that interval (only five values were above the mean + 2 SD in Bear B; all other values remained in the calculation). Increases in FGM levels after transport were considered biologically relevant when exceeding baseline concentrations by 2 SD (Vasconcellos *et al.*, 2011; Fanson *et al.*, 2017; Palme, 2019). We report the relative time after the transportation event at which FGM levels exceeded this individual threshold for the first time, when peaks appeared, and when FGM concentrations returned to values within 2 SD of the baseline.

#### Longitudinal measurements of faecal GC metabolites

To analyse the effect of season, sex and stress-inducing events (see Table 2) on FGM levels, a linear mixed-effects regression of log10-transformed FGM measurements was performed. Faecal samples were excluded from analysis if no sampling protocol was available for a sample or if the dates given on the sample bag and protocol were not consistent.

**Table 2:** Definition of explanatory variables tested for their effect on longitudinal faecal glucocorticoid metabolite measurement

Variable	Definition
Diarrhoea	Soft faeces (non-liquid)^a^
Sample age	Older/younger than 12 h^a^
Season:	
Pre-breeding	December to February
Breeding	March to May
Post-breeding	June/July
Non-breeding	August to November
Sex	Male/female
Stress-inducing events^b^:	
Environmental changes	e.g. new objects in enclosure/enrichment, change of enclosure
Social tension	Fight, mating, socialization, separation
Transport	Post-transport samples
Other disturbances	E.g. construction work, storm, fireworks, disease

^a^Based on records of sampling protocol

^b^Based on records of event log at days one to three before faecal sampling (according to a delay time of ~47 h until significant post-transport increase of FGMs, see Table 4)

The explanatory variable season was defined as a factor variable with four levels: breeding (March to May), non-breeding (August to November), pre-breeding (December to February) and post-breeding (June/July). Levels were based on a literature review of studies on captive as well as wild polar bear populations (Ramsay and Stirling, 1986; Amstrup and DeMaster, 2003; Puschmann *et al.*, 2009; Curry *et al.*, 2012a; Smith and Aars, 2015). Binary explanatory variables of different stress-inducing events (as derived from the sampling protocol or event log) were ‘social tension’, ‘transport’, ‘environmental changes’ and ‘other disturbances’. Moreover, diarrhoea and the age of faecal samples (older or younger than 12 h according to sampling protocol) were included as binary explanatory variables in the regression model to test their influence on FGM levels. For an overview and definition of all explanatory variables, see Table 2.

Model selection was based on the Akaike information criterion (AIC). The full model contained the main effect predictors (see Table 2) and the interactions between season and sex as well as between sex and social stress. Individual was included as a random effect, and an autoregressive model of order 1 (AR1) was used to account for temporal correlation. As recommended by Zuur *et al.* (2009), we first selected the optimal random effects structure based on restricted maximum likelihood estimation. The optimal fixed effects structure was selected based on maximum likelihood estimation and an exhaustive screening of all 416 candidate models up to the full model. Reported *P* values were based on likelihood ratio tests that compared the likelihood of the final model to the likelihood of the model for which the focal parameter was dropped. Residuals of the final model were visually examined for conforming to a normal distribution, and normalized residuals were inspected for temporal correlation and heteroscedasticity. All statistical analyses were performed in R, V3.5.1 (R Development Core Team, 2018). Linear mixed models for the log10-transformed FGM measurements were calculated using function lme of the nlme-package (Pinheiro *et al.*, 2019).

## Results

### GTT in polar bears

First coloured scats appeared on average 7.2 h after feeding dye markers. Shortest transit times (6.3 h, 6.5 h) resulted from feeding of large amounts of vegetable/fruits with egg white (in ice blocks) and small quantities of fish. Longer transit times (8 h, 7.8 h, 15 ± 7 h) were associated with the consumption of meat (including bones and fat). In one case, the male did not defaecate during the monitored time (after 8 h) and coloured scat was found the next morning (after 22 h, i.e. 15 ± 7 h)*—*transit times including and excluding this value are given in Table 3.

**Table 3:** Determination of gastrointestinal transit times by feeding dye markers to the usual morning feed of two polar bears

Polar bear	Sex	Age (years)	Appearance of first coloured scat (h after feeding)	
			Meat based diet^a^	Vegetarian diet^b^
I	m	15	8 (15 ± 7^c^)	6.3
J	f	25	7.8	6.5
Average transit time			7.9 (10.3^d^)	6.4
Total			7.2 (8.7^d^)	

^a^Including fat and bones

^b^Including small amounts of fish

^c^Defaecation 8–22 h after feeding

^d^Including 15 ± 7 h

### EIA validation (transports)

**Figure 1:**
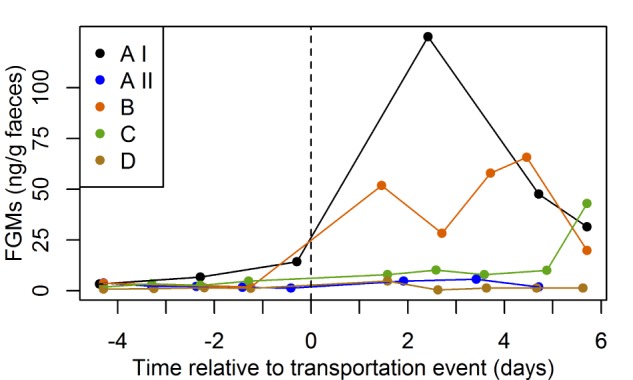
Individual faecal glucocorticoid metabolite levels (expressed as ng per g faeces) of four polar bears (A, B male; C, D female) during five transportation events in a 5-day time window before transport and at Days 1 to 6 after transport. Post-transportation levels were markedly increased compared to pre-transportation levels (*P* = 0.03) and there were significant inter-individual differences in pre-transportation levels (*P* = 0.02)

Median FGM levels 1 to 6 days after the transportation event were significantly elevated relative to FGM levels obtained over a 5-day window preceding the transportation event (one-sided paired Wilcoxon signed-rank test, *P* = 0.03; Fig. 1). Further, baseline FGM levels 5 days prior to the transportation events differed significantly between individuals (Kruskal–Wallis test, *P* = 0.02). When assessing individual values and temporal course, in all bears FGM concentrations exceeded the mean baseline +2 SD threshold within 3 days after a transportation event.

Taking into account the sampling time and the age of samples according to sampling protocols (samples older than 12 h were excluded, see discussion), FGM levels for all bears were higher than baseline +2 SD at a mean of 47 h after start of transport (i.e. time of loading). During that time, FGM levels were on average 7.8 times higher than individual baseline values (increases of 123–2013%). Individual FGM baseline values in the four transported polar bears ranged from 1.6 ± 0.7 to 6.1 ± 2.5 ng/g (mean ± SD; Table 4). Peak levels were reached 2 to 6 days (mean 88 h) after transportation, being up to 21.1 times higher than levels before transportation (peak increases of 163–2013%)*.* Detailed results for each bear are given in Table 4. Percentage increases of FGM levels 2 days after transportation relative to baseline levels of the two anaesthetized individuals (B: 750%, C: 286%) were within the range of values observed for the three non-anaesthetized individuals (123–2013%; Table 5). No consistent effect of transport length on the increase in FGM levels 2 days after transport was observed (Table 5).

**Table 4:** Faecal glucocorticoid metabolite levels of transported polar bears

PB	Sex	Baseline^a^ FGM conc pre-transport mean ± SD (ng/g) (samples/days)	First significant post-transport increase (> mean baseline +2 SD)			FGM peak			Return to baseline (< mean baseline +2 SD) (days after transport)	
			Conc (ng/g)	h after transport^b^ (± h range)	× times higher than baseline	Conc (ng/g)	h after transport^b^ (± h range)	× times higher than baseline	First	First of min 3 days in a row
A I	m	5.9 ± 4.3 (9/22)	125.2	±">62 ± 2	21.1	125.2	62 ± 2	21.1	13	33^c^
A II	m	2.2 ± 0.9 (6/5)	4.8	50 ± 4	2.2	5.7	86 ± 2	2.6	5	5^c^
B	m	6.1 ± 2.5 (25/52)	51.9	37 ± 2	8.5	65.7	108 ± 2	10.8	9	23
C	f^d^	2.1 ± 1.5 (9/8)	7.9	41.5 ± 2	3.9	43.0	141 ± 2^e^	21.0	7	ND^f^
D	f^d^	1.6 ± 0.7 (6/6)	4.8	42 ± 2	3.1	4.8	42 ± 2	3.1	3	3
Mean		3.6 ± 2.2	38.9 ± 52.2	46.5 ± 2.4	7.8	48.9 ± 49.9	87.8 ± 2	11.7	7.4	16

Abbreviations: conc, concentration; FGM, faecal glucocorticoid metabolite; min, minimum; PB, polar bear; SD, standard deviation

^a^Determined by an iterative approach: values above the mean + 2 SD were excluded (Palme, 2019)

^b^i.e. hours after start of loading

^c^>7 days between the three measurements

^d^Females presumably non-pregnant (see Table 1), not lactating or carrying cubs

^e^i.e. 86 ± 2 h after socialization with another female

^f^Not detected within monitored time

**Table 5:** Relationship between transport length, immobilization and percentage increase of faecal glucocorticoid metabolites at day two after transport (46.5 ± 2.4 h after start of loading, mean ± h range; see Table 4)

PB	Sex	Transport length (h)	Immobilization^a^	FGM increase above baseline^b^ (%)
A I	m	2.5	No	2013
A II	m	5.8	No	123
B	m	8	Yes	750
C	f	3	Yes	286
D	f	7.8	No	209

Abbreviations: FGM, faecal glucocorticoid metabolite; PB, polar bear

^a^For loading only

^b^Determined using an iterative approach (see Table 4)

FGM concentrations returned to values below baseline +2 SD for the first time on average 7 days (3–13 days) after transport. However, they did not remain at baseline values before a mean of 16 days (i.e. at least three consecutive days lower than baseline +2 SD). In one female, FGM levels did not return to baseline values for more than 2 days in a row within the total monitored time (15 days post transport; Table 4), despite daily food intake and regular defaecation and sampling (faecal material was only missing at Days 13 and 14 post-transport).

### Longitudinal measurements of faecal GC metabolites

**Figure 2:**
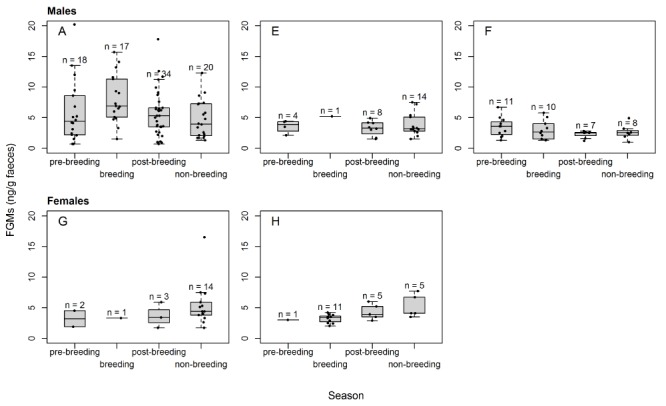
Box plot of faecal glucocorticoid metabolite levels (expressed as ng per g faeces) of five polar bears (A, E, F male; G, H female) in different seasons (pre-breeding: June/July; breeding: March–May; post-breeding: December–February; non-breeding: August—November). The number of data points underlying each box plot is indicated by n

The analysis of long-term FGM levels of five polar bears revealed that stress induced by social tension, environmental changes and other disturbances led to significant increases in FGM levels (Table 6). The strongest increases in FGM levels were observed for social stress, which led to a 4.7-fold rise above baseline FGM levels. Furthermore, measured FGM levels were significantly elevated (by a factor of 1.45) for faecal samples older than 12 h (*P* = 0.023). The transportation events of Bear A (Table 1) also led to significant rises in FGM concentrations (Table 6), as already described above (EIA validation). Season, sex and mild diarrhoea had no significant effect on FGM levels. However, we note that males tended to have higher FGM values during the breeding season from March to May as compared to the non-breeding season (Fig. 2). For the two females, the opposite pattern was observed with lower FGM concentrations during the breeding season compared to the non-breeding season (Fig. 2).

**Table 6:** Results of a linear mixed effects model testing the effect of different binary stress variables and sample age on log10-transformed faecal glucocorticoid metabolite measurements

	Effect size (on log10 (FGMs))	10^effect size	CI for 10^effect size	DF	Likelihood ratio	Pr (>Chi)
Intercept	0.58	3.79	3.00–4.79			
Social tension	0.67	4.70	2.55–8.67	1	234.54	0.001
Transport	0.42	2.61	1.41–4.81	1	92.40	0.002
Environmental changes	0.40	2.50	1.38–4.50	1	90.93	0.003
Other disturbances	0.50	3.17	2.10–4.88	1	262.04	<0.001
Sample age > 12 h	0.16	1.45	1.05–2.00	1	51.53	0.023

Reported *P* values are based on likelihood ratio tests that compare the likelihood of the final model to the likelihood of the model in which the focal parameter was dropped. The random effects structure controls for repeated measures per individual and temporal autocorrelation (using an AR(1) correlation structure). The estimated within-individual variation (σ2residual = 0.0997) exceeds the variation between individuals (σ2random intercept = 0.0098). The estimated AR(1) autocorrelation coefficient is *ρ* = 0.43. CI for 10^effect size gives 95% confidence intervals. Abbreviations: FGMs, faecal glucocorticoid metabolites

## Discussion

By measuring significantly higher GC metabolite concentrations in faeces of recently transported polar bears, we demonstrated that changes in the release of cortisol into the blood during a situation of stress directly affected their FGM levels, thus showing the biological validity of the used assay for this species (Palme, 2019). The EIA was able to detect FGMs in polar bears and therefore at least some of the excreted metabolites cross-reacted with the antibody of the applied cortisol assay. Even though it is not possible to predict which faecal metabolites may occur in a given species and a characterization of the specific polar bear FGMs has not been conducted (e.g. via HPLC separation (Schatz and Palme, 2001; Palme, 2019)), it was shown that the employed assay could trace fluctuations in metabolites that yield biologically relevant information relating to the bears’ HPA axis activity.

The average delay between transport related stress and clear FGM concentration increase was 47 h after loading the animals. This is consistent with previous work by Shepherdson *et al.* (2013), which demonstrated a marked FGM increase during the 48-h period of transport for two polar bears. In the same study, the highest FGM levels (16 times higher than baseline) were detected on Day 3 after ACTH injection of one polar bear (though no shorter intervals were sampled), which is similar to the current results (FGM peak levels average 11.7 times higher than baseline 2 to 6 days after transportation). However, in our study one juvenile female bear (C) was socialized with another young female 2 days after arrival at the new zoo. The corresponding FGM peak of this bear (86 h after socialization) could be due to socialization rather than transport related stress.

In accordance with previous work (Schatz and Palme, 2001; Palme *et al.*, 2005; Touma and Palme, 2005; Madliger and Love, 2014), we detected significant inter-individual differences in FGM levels during the 5-day window prior to transport. Inter-individual variation in cortisol concentrations could also be measured in hair (Bechshoft *et al.*, 2011; Bechshoft *et al.*, 2012; Bechshoft *et al.*, 2013) and plasma samples (Tryland *et al.*, 2002; Haave *et al.*, 2003) of free-ranging polar bears. Moreover, we observed that high inter-individual variability in measured peak FGM levels and reactivity to transport-related stress differed widely between the four bears (Table 4), which is consistent with comparable studies (Wells *et al.*, 2004; Palme *et al.*, 2005; Touma and Palme, 2005).

Percentage increases after zoo-to-zoo transports of two polar bears (717%, 480%) in a study conducted by Shepherdson *et al.* (2013) were similar to those observed in the current study. Furthermore, ACTH challenges revealed similar increases in FGMs in polar bears (1541%, *n* = 1; Shepherdson *et al.*, 2013), polar and grizzly bears (343–2258%, *n* = 6; White *et al.*, 2015) and several other carnivores including Himalayan black bear (401% and 573%, measured via cortisol EIA and corticosterone RIA, respectively; Young *et al.*, 2004).

Even so, in our study pre-transport baseline levels were much lower compared to the average 3-day range of zoo polar bears before transport or ACTH test as reported by Shepherdson *et al.* (2013; 175.5 ng/g, *n* = 2 and 256 ng/g, *n* = 1, respectively) or captive polar bear baseline FGM concentrations established by White *et al.* (2015; 33.8 ± 9 ng/g; mean ± SEM, *n* = 3) and Bryant and Roth (2018; six females: 29.98 ± 11.40 ng/g; mean ± SD). However, this is likely due to the different applied assays (two different corticosterone RIAs and two cortisol EIAs were used) and their antibody affinity for different metabolites (White *et al.*, 2015; Palme, 2019). This underscores the need to employ consistent sampling and analytical approaches in such studies in order to be able to directly compare results.

One crate trained male (trained to enter the transport box using positive reinforcement; Individual A) was sampled over two transports and showed clear differences in reactivity to transport related stress (peak levels 21 and 3 times higher than baseline). However, baseline values were in the same range when comparing both data sets (Table 4). Intra-individual differences in the release of GCs have also been described for polar bears by Bryant and Roth (2018; not fully validated method). Besides natural individual variation, different seasons or the advanced training level in the second transport could be possible explanations (see below). Only few studies exist that investigate the influence of positive reinforcement training on GCs, suggesting that trained animals cope better with situations of stress. Such animals showed calmer behaviour than untrained conspecifics during blood sampling or ultrasound examination, and their GC levels did not rise (Phillips *et al.*, 1998; Grandin, 2000; Capiro *et al.*, 2014, whereby only the first study provides values before training). We observed that transport events were linked to a rapid, but temporary increase in FGMs in polar bears, which reflected an intact and adequate stress response. Elevated FGM concentrations first returned to values below the threshold of baseline +2 SD after an average period of 7 days after transport. For polar bears, a return to near baseline levels is described for the 3-day range beginning 2 days after transport; however, no faecal samples were collected at a later time (Shepherdson *et al.*, 2013). The observation that FGMs remained at baseline values after about 2 weeks (Table 4) indicated that such necessary transportations are only a short-lasting stressor, and bears quickly adapt to the new environment. This is important because coordinated breeding efforts of EAZA accredited zoos are crucial to maintaining a demographically and genetically stable population, which is the main goal of the polar bear long-term management plan of the European Endangered Species Programme (EEP; Szánthó and Schad, 2014). Although we could not find an effect of transport length or immobilization on FGM levels, we also cannot rule out an impact due to the low number of monitored individuals. We suggest to keep transport duration as short as possible (for a review on farm animals, see Nielsen *et al.*, 2011) and to choose positive reinforcement training over anaesthesia for loading to minimize possibly associated negative effects (Grandin, 1997; Capiro *et al.*, 2014).

During longitudinal measurements, the stressors social tension, environmental changes and other disturbances led to significantly higher FGM levels in captive polar bears (besides transport). This highlights the necessity to include external factors whenever assessing FGMs of an individual. Considering that ‘positive stressors’ (e.g. environmental enrichment, mating; see Broom, 1988; Möstl and Palme, 2002; Capiro *et al.*, 2014) were included as explanatory variables and also resulted in short-term increases of FGMs, one must be careful with interpretation of elevated FGM levels. Thus, a distinction of different reasons for stress responses might be necessary when evaluating increases in FGMs, especially in the context of animal welfare assessment. The duration of elevated FGM concentrations needs to be kept in mind in this context: positive and negative stimulation can lead to an acute activation of the HPA axis, which enables the individual to cope with a new situation and is therefore essential. However, as stated above, prolonged HPA axis activity and resulting chronically elevated cortisol levels have been linked to reduced well-being, immunosuppression, impaired growth and reproduction rates in many species (Sapolsky *et al.*, 2000; Habib *et al.*, 2001; McEwen and Wingfield, 2003; Romero *et al.*, 2009).

FGM values were significantly elevated in faeces older than 12 h, thus underscoring the importance of a standardized sampling protocol, thorough selection and rapid freezing of fresh faecal samples (Millspaugh and Washburn, 2004; Palme, 2005; Touma and Palme, 2005).

We could not identify a significant effect of sex or season on polar bear FGM levels when applying the pre-defined breeding seasons. However, the sample size of only three males and two females is too small to draw strong conclusions. To the best of our knowledge, no study assessing both male and female polar bear long-term FGM levels has been performed before, thus no comparison with our results is possible at this point. According to the study by Bryant and Roth (2018), where faeces from six zoo-housed female polar bears were sampled for 12 consecutive months and tested for FGM levels, no seasonal differences in FGM concentrations could be detected. Nevertheless, a seasonal influence of the HPA axis reactivity is described for many seasonally breeding vertebrates including black bears (Harlow *et al.*, 1990; Wingfield and Romero, 2010; Romero, 2002; Millspaugh and Washburn, 2004; Jachowski *et al.*, 2015). In addition, reproductive status in both sexes (testes active or not; non-reproductive, pregnant, lactating) is presumed to affect mammalian blood GC levels and should be reflected in FGMs as well (Millspaugh and Washburn, 2004; Sheriff *et al.*, 2010; Palme, 2019). However, due to the unknown reproductive status of some animals and the low number of individuals, we could not investigate such an influence. Our longitudinally monitored females were presumed to be non-pregnant, but we cannot rule out pregnancy with certainty, because delayed implantation, pseudopregnancy and the lack of an accurate and non-invasive pregnancy test for polar bears make a reliable diagnosis difficult (Curry *et al.*, 2012b; Stoops *et al.*, 2012).

However, season could be a possible explanation for the differing FGM increases after the two monitored transports of Polar Bear A (AI and II; Table 4): the percentage rise of FGMs was 17 times higher after translocation in March, which is the beginning of the breeding season, compared to the percentage increase after translocation in November of the same year, which falls within the time of low or no sexual activity in polar bears (Ramsay and Stirling, 1986; Amstrup and DeMaster, 2003; Puschmann *et al.*, 2009; Curry *et al.*, 2012a). In both cases, sexually mature females have been present in the destination zoo, olfactorily or/and visually noticeable for the male (which was the only monitored male that has successfully bred before, Table 1).

Our measured GTTs and the fact that a mainly vegetarian diet shortened GTT resemble the results Pritchard and Robbins (1990) determined in grizzly and black bears: in both species, GTT of a vegetarian diet (7 h) was nearly half that of a meat diet (13 h). Similar results for polar bears were presented by Best (1985) for meat diets (including fish; 12.3–18.6 h), whereas Shepherdson *et al.* (2013) indicated an average gut passage time of ~24 h for polar bears (type of diet not specified). These findings demonstrate an influence of diet on GTTs and therefore on the appearance of peak FGM levels following a stressor. We found longer delay times in the monitored transports, which may indicate that GTTs are less appropriate for assessing the time delay of blood GC levels to an increase of FGMs. However, one needs to bear in mind that in the mentioned GTT studies including ours, first appearance of dye (or mean retention times) and not peak excretion were determined. In addition, transportation is a complex stressor, and plasma peak GC levels most likely occurred to the end of the transportation (3–8 h), or even afterwards, when the bears experienced additional stressors due to the new environment. This may explain the longer delay times observed in our study. Still, our recorded GTTs (time until first appearance of dye) are useful as they indicate the interval in which baseline values can still be assumed with certainty. For capturing peak levels with great probability, frequent sample collection is required (Palme, 2019). Finally, to empirically determine the whole duration of cortisol metabolism and excretion in polar bears, radiometabolism studies would need to be conducted (Schatz and Palme, 2001; Keckeis *et al.*, 2012).

The factors season and diet are especially important with regard to FGM monitoring in wild polar bears: composition and amount of food intake highly vary in free-ranging polar bears due to differing availability or accessibility of food throughout the year. Polar bears undergo long periods of ‘fasting’ during the summer months and early fall (only occasional feeding on berries/vegetation, birds’ eggs, geese, caribou, carcasses or small mammals) before a phase of increased food intake in winter and spring (main food source ringed (*Phoca hispida*) and bearded seals (*Erignathus barbatus*); Brook and Richardson, 2002; Derocher *et al.*, 2002; Bentzen *et al.*, 2007; Thiemann *et al.*, 2008; Gormezano and Rockwell, 2013; Iversen *et al.*, 2013). Pregnant females remain fasting in their den from fall until spring, when they emerge with their cubs (Amstrup and DeMaster, 2003; Stirling, 2012). The temporarily high proportion of fat and blubber in the diet of free-ranging polar bears is another unique characteristic (Stirling, 1974; DeMaster and Stirling, 1981; Best, 1985) and differs from captive polar bears diet with higher amounts of meat, fish, commercial dog food, fruit, vegetables and leftovers of human food (authors’ personal experience and results of questionnaire, see Study animals and facilities; Lintzenich *et al.*, 2006). However, seasonal fluctuations of the diet and especially of the actual consumption of food can also be observed in captive polar bears. According to the authors’ personal experience, polar bear keepers of several participating zoos (T. Ramm, M. Ehlers, Karlsruhe Zoo; S. Krüger, Nuremberg Zoo; J. Bartunek, Vienna Zoo) and Lintzenich *et al.* (2006), food intake of polar bears is less during the summer months (around late May to September) compared to the rest of the year—regardless of the fact that sufficient amounts of different food items were offered in all zoos. Differences in the composition of the diet, frequency of feeding and defaecation as well as digestion type can highly influence the amount and distribution of measured FGMs (Palme, 2005; Palme *et al.*, 2005; Touma and Palme, 2005; Sheriff *et al.*, 2011; Ganswindt *et al.*, 2012; Palme, 2019) and should therefore be taken into account in future FGM studies on polar bears.

Besides all the abovementioned external factors, a natural individual variation remains that can strongly affect GC levels. These inter-animal differences in GC concentrations and differing responses to stimuli also depend on individual factors like genetics, age, physiological condition and previous experience (Grandin, 1997; Moberg, 2000). Individual characteristics of zoo bears can be taken into account for example by assessing ‘life history’ and ‘temperament’ of an individual as suggested by Shepherdson *et al.* (2013): captive polar bears that scored high on the ‘interest’ axis (defined in relation to behaviour directed toward a newly introduced object) exhibited lower FGM levels over a 1-year period. In our study, ‘transport experience’ was factored in as an element of ‘life history’: two of the four transported bears had been transported before (A, B; Table 1). Due to the mentioned natural inter-individual variability of GC baselines and individual reactivity to stress, a direct comparison of experienced and non-experienced animals is not useful. Nevertheless, in Male A, who was monitored during two transports, habituation and a learning effect could be possible additional reasons for the much lower peak observed after the second transport (Tables 1 and 4). Arguing against this is the fact that this male bear had also been transported before the first monitored transport in March, even if it was almost 5 years prior (Linke, 2016).

Extensive behavioural observations were neither possible nor the focus of this study; thus, only specific pre-defined events and behaviours were recorded. Captive polar bears are particularly prone to repetitive behaviours like pacing (Poulsen *et al.*, 1996; Shepherdson *et al.*, 2004; Swaisgood and Shepherdson, 2005; Cremers and Geutjes, 2012; Shepherdson *et al.*, 2013). In future FGM and welfare assessment studies, the occurrence, extent and type of stereotypies (underlying motivation; see Mason and Latham, 2004) and their development under environmental changes should be included, since their relation to FGM levels is not entirely clear (Shepherdson *et al.*, 2013: higher FGM levels linked to higher proportions of stereotypic pacing vs. Shepherdson *et al.*, 2004: higher peak FGM concentrations and higher FGM variation in non-stereotyping bears; for a review on stereotypies as an indicator of welfare, see Mason and Latham, 2004).

Measuring FGM levels could contribute significantly to a holistic approach to welfare assessment of zoo polar bears. In conjunction with behavioural and other physiological markers (e.g. body condition, blood values), the health and well-being of bears can be evaluated under various influences like different enclosure features (e.g. number and type of hiding places, view out of exhibit, proportion of natural soil), group composition (sex, age, reproductive state, number of individuals) or managing details (e.g. environmental enrichment, positive reinforcement training, freedom of choice (e.g. inside/outside enclosure), predictability of feeding times) (Yeates and Main, 2008; Shepherdson *et al.*, 2013; Rose *et al.*, 2017; Bacon, 2018).

For the application of FGM monitoring in the field, the varying food intakes of wild polar bears can be factored in by differentiating between samples collected during onshore fast vs. phases of hyperphagia (e.g. comparing samples over several summers or winters, respectively). Furthermore, food items can be accessed via faeces (von der Ohe *et al.*, 2004; Bentzen *et al.*, 2007; Iversen *et al.*, 2013) and thus their potential influence on FGM levels taken into account. All in all, analysing FGMs has great potential also for wild bears as it completes the wide range of already established non-invasive analyses of faeces (like DNA, population size, reproductive status, contaminant load, parasite infestation, demographic and life-history data; e.g. Kohn and Wayne, 1997). One of the most important concerns to polar bear health is climate change (Stirling and Derocher, 1993; Stirling *et al.*, 1999; Stirling and Parkinson, 2006; Wiig *et al.*, 2008; Patyk *et al.*, 2015), which is particularly significant in the Arctic (Stroeve *et al.*, 2007; Bernstein *et al.*, 2008). Even though polar bears periodically experience nutritional stress in times of seasonal food scarcity or reproductive fasting (Hamilton, 2008; Mislan *et al.*, 2016), the rapidly progressing environmental changes and other ecological, biological and social factors (e.g. increased exposure to contaminants and emerging diseases, human interactions or exposure to competitors) lead to a cumulative effect that exceeds the natural periodical experiences. Finally, there are numerous studies providing evidence that polar bears cannot cope long-term with this variety of mutually reinforcing stressors (Derocher *et al.*, 2004; ACIA, 2005; Molnár *et al.*, 2010; Stirling and Derocher, 2012; Bechshoft *et al.*, 2013). Besides the characterization and examination of the different impacts on polar bear health, a systematic and standardized monitoring and data collection is vital to compare across populations and to enable a holistic approach to a circumpolar conservation effort (Patyk *et al.*, 2015). Together with parameters as body condition, sea ice condition or reproductive success, measuring FGM levels can aid to evaluate population health status and thus possible consequences under current climate trends investigated.

We validated a cortisol EIA for measuring FGM concentrations in polar bears. Furthermore, in this study for the first time FGM levels of both female and male polar bears were monitored for a prolonged period of time in European zoos, taking also cause–effect relations into account. The results of our research provide basic information on polar bear endocrinology, GTTs and factors influencing HPA axis activity. Social tension, transport, environmental and other external changes resulted in an acute increase in FGM levels in polar bears and therefore need to be taken into account when assessing long-term HPA axis activity in these animals*.* We highly recommend the use of a simple but detailed sampling protocol including diet, pre-defined external factors and sample age (faecal material older than 12 h should be excluded from analysis). Significant intra- and inter-individual differences in baselines and stress responses were observed, preventing the establishment of general polar bear GC reference values or thresholds marking stress. Instead, individual GC profiles should be determined by assessing FGM levels over prolonged sampling periods of the same bear. Further investigation of individual FGM baseline values and circannual or nutritional fluctuations in captive polar bears is essential for the interpretation and understanding of FGM levels from wild bears, since a validation and establishment of baselines would be difficult under field conditions. Even though a narrow definition of GC reference values for polar bears is currently not possible, basic information on polar bear endocrinology and long-term monitoring of GC levels will be useful for managing polar bears in captivity and for monitoring stress in wild polar bears faced with degradation of their habitat.

## Supplementary Material

Supplementary_coaa012
