# Supplementary material for: Faecal glucocorticoid metabolites as a measure of adrenocortical activity in polar bears (Ursus maritimus)
Source: Conserv Physiol. 2020 Apr 4;8(1):coaa012. doi: 10.1093/conphys/coaa012 (PMC7125046; doi:10.1093/conphys/coaa012)
Supplement: Supplementary_coaa012 [file supplementary_coaa012.zip › Supplementary_coaa012/Heinetal_Polar_Bears_FGM_SupplementaryData_II_coaa012.docx]

**Supplementary Material II**

**Faecal Glucocorticoid Metabolites in Polar Bears (*Ursus maritimus*) as a measure of adrenocortical activity**

*Anna Hein, Rupert Palme, Katrin Baumgartner, Lorenzo von Fersen, Benno Woelfing, Alex D. Greenwood, Thea Bechshoft, Ursula Siebert*

**Questionnaire**

Name of surveyed facility:

…………………………………………………………………………………………………...…………………………………………………………………………………………………...…………………………………………………………………………………………………………………………...

Answered by:

…………………………………………………………………………………………………...

Contact person polar bear district:

………………………………………………...…………………………………………………

Phone.: ………………………………….. Email: ………………………………………...……

Note: The questionnaire will be completed together during the interview.

Therefor details on enclosure size, breeding, feeding lists etc. should be kept available.

**I. Individual animal data**

1.) How many polar bears (PB) are being kept in your facility at the moment?

….. ♂ ….. ♀

2.) Name, age, sex and background? Since when housed in the current zoo?

| Name PB | Date of birth | ♂/♀ | Place of origin/zoo | Parents (zoo) | since |
| --- | --- | --- | --- | --- | --- |
|  |  |  |  | M:  F: |  |
|  |  |  |  | M:  F: |  |
|  |  |  |  | M:  F: |  |
|  |  |  |  | M:  F: |  |
|  |  |  |  | M:  F: |  |

**II. Husbandry details**

1.) Since when have you been keeping polar bears in your facility?

…………………………………………………………………………………………………….

2.) Since when has the current polar bear enclosure been in use?

…………………………………………………………………………………………………….

3.) How big are the enclosures? (area in m²)

| Outdoor enclosure name/no. | Size |
| --- | --- |
|  |  |
|  |  |
|  |  |
|  |  |

| Indoor/stall name/no. | Size |
| --- | --- |
|  |  |
|  |  |
|  |  |
|  |  |
|  |  |

Is there visual contact between the enclosures/stalls?

………………………………………………………………………………………………..…………………………………………………………………………………………………..…………………………………………………………………………………………………..……………………………………………………………………………………………………..

4.) Details on enclosure structure/substrate/vegetation (enclosure sketch):

………………………………………………………………………………………………….…………………………………………………………………………………………………….…………………………………………………………………………………………………….…………………………………………………………………………………………………….....................................

5.) How many polar bears are kept per enclosure? Predominant utilization of the enclosure

(> 6 months per year)

| Enclosure name/no. | Number of PB | | Names of PB |
| --- | --- | --- | --- |
|  | ♂ | ♀ |  |
|  |  |  |  |
|  |  |  |  |
|  |  |  |  |
|  |  |  |  |

**III. Management**

1.) Daily husbandry routines (stable times, feeding, show, training etc.)?

…………………………………………………………………………………………………….…………………………………………………………………………………………………….…………………………………………………………………………………………………….…………………………………………………………………………………………………….………………………………………………………………………………………………….…………………………………………………………………………………………………….……………………………………………………………………………………………………….

2.) How often are the bears fed? When (daytime)?

………………………………………………………………………………………………….…………………………………………………………………………………………………….……………………………………………………………………………………………………….

3.) What are you feeding? Any seasonal changes? (Feeding list)

………………………………………………………………………………………………….…………………………………………………………………………………………………….…………………………………………………………………………………………………….…………………………………………………………………………………………………..…...…………………………………………………………………………………………………….

4.) Method of feeding (scattered/hidden food, outside feeding, separated indoor feeding)?

………………………………………………………………………………………………….….………………………………………………………………………………………………….…….……………………………………………………………………………………………….………………..…

5.) Has there been successful breeding within the last 5 years (in current enclosure)?

| Offspring (mother) | Date of birth | Hand-rearing/Mother | | Current enclosure | | P.r.n. date of death |
| --- | --- | --- | --- | --- | --- | --- |
|  |  | H | M | Yes (No.) | No |  |
|  |  |  |  |  |  |  |
|  |  |  |  |  |  |  |
|  |  |  |  |  |  |  |
|  |  |  |  |  |  |  |
|  |  |  |  |  |  |  |

6.) Administration of medicaments, food supplements etc.?

……………………………………………………………………………………………….…………………………………………………………………………………………………….……………………………………………………………………………………………………….……………………………………………………………………………………………………….

7.) Are there enrichments? What sort of? How often?

| Type of enrichment | Day-to-day | Static | Animal acceptance  (1= low, 5= high) | | | | |
| --- | --- | --- | --- | --- | --- | --- | --- |
|  | Frequency |  | 1 | 2 | 3 | 4 | 5 |
|  |  |  |  |  |  |  |  |
|  |  |  |  |  |  |  |  |
|  |  |  |  |  |  |  |  |
|  |  |  |  |  |  |  |  |
|  |  |  |  |  |  |  |  |
|  |  |  |  |  |  |  |  |

Any further remarks:

.………………………………………………………………………………………………..…...……………………………………………………………………………………………………………………………………………………………………………………………………...…………………………………………………………………………………………………...………………………………………………………………………………………………..…..………………………………………
