# Supplementary material for: Faecal glucocorticoid metabolites as a measure of adrenocortical activity in polar bears (Ursus maritimus)
Source: Conserv Physiol. 2020 Apr 4;8(1):coaa012. doi: 10.1093/conphys/coaa012 (PMC7125046; doi:10.1093/conphys/coaa012)
Supplement: Supplementary_coaa012 [file supplementary_coaa012.zip › Supplementary_coaa012/Heinetal_Polar_Bears_FGM_SupplementaryData_I_coaa012.docx]

**Supplementary Material I**

**Faecal Glucocorticoid Metabolites in Polar Bears (*Ursus maritimus*) as a measure of adrenocortical activity**

*Anna Hein, Rupert Palme, Katrin Baumgartner, Lorenzo von Fersen, Benno Woelfing, Alex D. Greenwood, Thea Bechshoft, Ursula Siebert*

**1) Faecal sampling protocol**

Name of polar bear: …..…………………..…………… Zoo: ……………………………………………..

| Sample-no. (continuous) | Sampling date | Sampling time | Sample freshness  (**e**stimated/ **k**nown) in h | | | | Enclosure name/no. | Weather conditions  (**w**et **r**ain/**s**now, **d**ry; faeces from **b**ox) | | | | Temperature (°C) | | | | | Faeces consistency (**s**oft/**d**iarrhoea; **n**ormal) | | Miscellaneous  (medication, gestation, disease,  social stress etc.) |
| --- | --- | --- | --- | --- | --- | --- | --- | --- | --- | --- | --- | --- | --- | --- | --- | --- | --- | --- | --- |
|  |  |  | e | | k | |  |  |  |  |  |  |  |  |  |  |  |  |  |
|  |  |  | <8 | 8  -  12 | | 12  -  24 |  | wr | ws | d | b | < 0 | 0  - 10 | 10 - 20 | 20 - 25 | > 25 | s/ d | n |  |
|  |  |  |  | |  | |  |  |  |  |  |  |  |  |  |  |  |  |  |
|  |  |  |  |  | |  |  |  |  |  |  |  |  |  |  |  |  |  |  |
|  |  |  |  | |  | |  |  |  |  |  |  |  |  |  |  |  |  |  |
|  |  |  |  |  | |  |  |  |  |  |  |  |  |  |  |  |  |  |  |
|  |  |  |  | |  | |  |  |  |  |  |  |  |  |  |  |  |  |  |

**2) Event log**

Name of polar bear: ………….……. Month/Year: ………..……./…..…… Enclosure: ……...... Solitary housing: yes O no O BCS: …....

|  | Translocation  (to enclosure) | | Socialisation | | Separation | | Fight | | Mating | | Disease | | Environ. changes | | other possible stressful events/ comments |
| --- | --- | --- | --- | --- | --- | --- | --- | --- | --- | --- | --- | --- | --- | --- | --- |
|  | yes | no | yes | no | yes | no | yes | no | yes | no | yes | no | yes | no |  |
| 01. |  |  |  |  |  |  |  |  |  |  |  |  |  |  |  |
| 02. |  |  |  |  |  |  |  |  |  |  |  |  |  |  |  |
| 03. |  |  |  |  |  |  |  |  |  |  |  |  |  |  |  |
| 04. |  |  |  |  |  |  |  |  |  |  |  |  |  |  |  |
| 05. |  |  |  |  |  |  |  |  |  |  |  |  |  |  |  |
| 06. |  |  |  |  |  |  |  |  |  |  |  |  |  |  |  |
| 07. |  |  |  |  |  |  |  |  |  |  |  |  |  |  |  |
| 08. |  |  |  |  |  |  |  |  |  |  |  |  |  |  |  |
|  |  |  |  |  |  |  |  |  |  |  |  |  |  |  |  |

BCS: Body Condition Score- according to Body Condition Index by Polar Bears International

**Definition of events**

| Event | Definition |
| --- | --- |
| Translocation | Change of enclosure (e.g. from big enclosure to small enclosure) |
| Socialisation | Change from individual housing to group housing; new animal in group |
| Separation | Change from group housing to individual housing |
| Fight | Conflict with conspecific(s) |
| Mating | Copulation |
| Disease | E.g. pododermatitis, diarrhoea |
| Environmental changes | Installation/removal of fixed objects in the enclosure (excl. day-to-day enrichments);  construction works/noise etc. in/near the enclosure |
